# Double-edged effects caused by magnesium ions and alkaline environment regulate bioactivities of magnesium-incorporated silicocarnotite *in vitro*

**DOI:** 10.1093/rb/rbab016

**Published:** 2021-09-02

**Authors:** Qiang Wu, Shunxiang Xu, Fei Wang, Bo He, Xin Wang, Ye Sun, Congqin Ning, Kerong Dai

**Affiliations:** 1 Shanghai Key Laboratory of Orthopedic Implant, Department of Orthopaedic Surgery, Shanghai Ninth People’s Hospital, Shanghai Jiao Tong University School of Medicine, No. 639, Zhizaoju Road, Shanghai, Huangpu District 200011, China; 2 The Education Ministry Key Lab of Resource Chemistry and Shanghai Key Laboratory of Rare Earth Functional Materials, Shanghai Normal University, No. 100, Guilin Road, Shanghai, Xuhui District 200234, China; 3 Department of Orthopedics Trauma and Microsurgery, Zhongnan Hospital of Wuhan University, No.169, East Lake Road, Wuchang District, Wuhan 430071, China; 4 Department of Orthopaedics, The First Affiliated Hospital of Nanjing Medical University, No.300, Guangzhou Road, Drum-tower District, Nanjing, 210029, China; 5 State Key Laboratory of High Performance Ceramics and Superfine Microstructure, Shanghai Institute of Ceramics, Chinese Academy of Sciences, No.1295, Dingxi Road, Changning District, Shanghai 200050, China

**Keywords:** silicocarnotite, magnesium, double-edged effects, osteogenesis and angiogenesis

## Abstract

Magnesium (Mg) is an important element for its enhanced osteogenic and angiogenic properties *in vitro* and *in vivo*, however, the inherent alkalinity is the adverse factor that needs further attention. In order to study the role of alkalinity in regulating osteogenesis and angiogenesis *in vitro*, magnesium-silicocarnotite [Mg-Ca_5_(PO_4_)_2_SiO_4_, Mg-CPS] was designed and fabricated. In this study, Mg-CPS showed better osteogenic and angiogenic properties than CPS within 10 wt.% magnesium oxide (MgO), since the adversity of alkaline condition was covered by the benefits of improved Mg ion concentrations through activating Smad2/3-Runx2 signaling pathway in MC3T3-E1 cells and PI3K-AKT signaling pathway in human umbilical vein endothelial cells *in vitro*. Besides, provided that MgO was incorporated with 15 wt.% in CPS, the bioactivities had declined due to the environment consisting of higher-concentrated Mg ions, stronger alkalinity and lower Ca/P/Si ions caused. According to the results, it indicated that bioactivities of Mg-CPS *in vitro* were regulated by the double-edged effects, which were the consequence of Mg ions and alkaline environment combined. Therefore, if MgO is properly incorporated in CPS, the improved bioactivities could cover alkaline adversity, making Mg-CPS bioceramics promising in orthopedic clinical application for its enhancement of osteogenesis and angiogenesis *in vitro*.

## Introduction

Magnesium (Mg) is an important element for human beings to maintain homeostasis, including stabilizing DNA, regulating ion channels on cell membranes and remaining physiological functions of bones [1–3]. Coupled with its biodegradability and similar elastic modulus of cortical bone, Mg has widely been incorporated into biomaterials aimed at facilitating bone regeneration [4, 5]. Over the past years, it has been confirmed that Mg promotes osteogenesis by proliferating osteoblasts, enhancing osteogenic differentiation and triggering mineralization *in vitro* [3, 6, 7]. Along with the satisfying outcomes of Mg screws applied in goats with femoral neck fracture and in patients with osteonecrosis of femoral head [8, 9], Mg has solidly verified its indispensable role in osteogenesis. On the other hand, in the course of bone regeneration, ensuring angiogenesis is also pivotal because successful osteogenesis is dependent on nutrients and oxygen’s delivered by locally restored blood supply [10]. This goal could also be achieved by Mg for its modulating microvascular functions *in vivo* [11]. Specifically, Mg-doped materials have proven to own enhanced angiogenic property in recent years, such as stimulating the production of reactive oxygen species, upregulating immune responses or promoting VEGF expression in human endothelial cells [12–14]. As a result, they have also been applied *in vivo* and achieved satisfying outcomes of vessel formation in animal models [15, 16]. Considering the merits of facilitating osteogenesis and angiogenesis combined, Mg is promising for improving bioactivities of biomaterials.

However, the side effects of biodegradable Mg cannot be overlooked. As pure Mg dissolves in culture medium, it generates hydrogen and magnesium hydroxide [Mg(OH)_2_], resulting in pH increase and alkaline environment [17]. The alkaline pH caused by pure Mg degradation is cytotoxic *in vitro*, including inhibiting cell adherence and proliferation [18, 19]. Even in Mg-contained alloy, cell hemolysis and cytotoxicity will occur if pH passes certain scope [20, 21]. Considering such disadvantages of Mg-contained metals, incorporating magnesium oxide (MgO) into biodegradable bioceramics may be a feasible way to exploit the merits of Mg while avoiding sharp pH change. First, the product of MgO reacting with culture medium or body fluids only includes Mg(OH)_2_, without generating hydrogen like pure Mg did. Second, Mg or Mg-contained alloys are vulnerable to corrosion resistance and therefore yield drastic pH shift when soaked in liquid [4, 17, 22], however, inorganic bioceramics do not react with culture medium directly, as Mg-containing bioceramics slowly degrade over time, Mg will release tenderly in Mg^2+^ form, avoiding acute pH rise. Together with the facts that the proper concentration change of Mg^2+^ in culture medium has no cytotoxic effects on cells [18, 20], it is reasonable to incorporate proper dosage of MgO into bioceramics to improve bioactivities. The assumption is indeed corroborated via CaO-MgO-SiO_2_-based bioceramics including akermanite, bredigite and diopside, the extracts of which could promote proliferation of bone mesenchymal stem cells and human umbilical vein endothelial cells (HUVECs) [23]. The osteogenic mechanisms of CaO-MgO-SiO_2_ composite bioceramics contain improved apatite formation ability as well as increased intake of ions amongst osteoblast-like cells in extracts [24–26]. Moreover, increased MgO contents in merwinite, akermanite or monticellite ceramics have all resulted in better adherence and spread of osteoblasts on ceramic wafers [27]. Therefore, MgO is an efficient dopant for bioceramics aimed at facilitating osteogenesis and angiogenesis.

Silicocarnotite [Ca_5_(PO_4_)_2_SiO_4_, CPS] bioceramics is an emerging candidate of orthopedic materials. Compared with conventional calcium phosphates like hydroxyapatite (HA), CPS ceramics have better performances of cytocompatibility and solubility [28]. Besides, CPS ceramics have demonstrated superior osteogenic property to HA *in vitro* and *in vivo* [29, 30]. Even though the mechanical property of CPS is not satisfying, the drawback could be overcome by adding elements [31]. In the meantime, doping elements is also an efficient way to improve the inherent bioactivities of CPS. For example, strontium-substituted CPS ceramics are able to enhance osteoblastic activities and inhibit osteoclastic behaviors, implying the potential application in the treatment of osteoporotic bone defect [32]. Considering the verified merits of Mg mentioned above, it is sensible to deduce that incorporating MgO into CPS (Mg-CPS) is another reliable way to possibly facilitate osteogenic and angiogenic properties.

In our study, CPS doped with different mass fractions of MgO (0/5/10/15 wt.%) were designed. When preparing extracts by soaking Mg-CPS powers in culture medium, varied concentrations of elements generated in extracts. By culturing mouse pre-osteoblasts (MC3T3-E1 cells) and HUVECs within Mg-CPS extracts, cell bioactivities were detected and analysed on gene, protein and cell levels. Particularly, osteogenic pathway of Smad2/3-Runx2 was detected for its significance of triggering osteogenic differentiation [33]. Besides, in order to study signaling changes of angiogenesis, PI3K-AKT was explored due to its vital role in vessel formation [34]. By analysing the activities of phosphorylation, we hope to elucidate the possible mechanisms under the influence of Mg-CPS extracts. Our study illustrated that even though alkaline circumstance of extracts strengthened with MgO addition, osteogenic and angiogenic activities of cells boosted in extracts with increased Mg^2+^ concentration provided that CPS was doped within 10 wt.% MgO. Consequently, as long as MgO is appropriately incorporated into CPS, the side effect of causing alkalinity could be offset by its enhanced bioactivities, and Mg-CPS is a promising biomaterial in orthopedic application for its improved osteogenic and angiogenic abilities.

## Materials and methods

### Materials preparation and characterization

CPS powders were synthesized by the sol-gel method as reported previously [28], and MgO powders (50 nm, 99.9% and sphere) were purchased from Shanghai Macklin Scientific Co., Ltd, China. Morphology of both raw materials was shown in Supplementary Fig. S1. Briefly, a series amount of MgO (0/5/10/15 wt.%) powders were added to CPS, followed by ball-mixing with ethyl alcohol for 5 h. The mixed powders were dried in the oven at 60°C for 24 h and then pressed into a stainless steel mold for preforming, followed by a cold isostatic pressing at 250 MPa for 5 min. Finally, the green bodies were sintered in a muffle furnace at 1300°C for 2 h to obtain Mg-CPS bioceramics. Four kinds of Mg-CPS specimens were called briefly as CPS, 5 Mg-CPS, 10 Mg-CPS and 15 Mg-CPS, respectively.

Phase composition and functional groups of Mg-CPS bioceramic powders was characterized by X-ray diffraction (XRD, D/MAX-RBX, Rigaku, Japan) and Fourier transform infrared spectroscope (FTIR, IRAffinity-1, Shimadzu, Japan). Microstructure of which was observed with scanning electron microscopy (SEM, Model S-3400N, Hitachi, Japan). Element mapping of 15 Mg-CPS specimen was investigated by a field emission scanning electron microscope (SU8220, Hitachi, Japan) equipped with an energy dispersive X-ray spectrometer. To analyse the sinterability of Mg-CPS bioceramics with different MgO content, relative density (RD) and linear shrinkage (LS) were computed. For RD, the theoretical density (*ρ*_0_) of Mg-CPS bioceramics was calculated, respectively, according to the formula: *ρ*_0_ = *ρ*_(MgO)_ * × wt.% MgO + *ρ*_(CPS)_ * *y* wt.% CPS, actual density (*ρ*) was calculated by *ρ* = *m*/*v* = *m*/(*πr*^2^*h), then RD was calculated by RD = *ρ*_/_*ρ*_0_ (*m*, *r* and *h* represent mass, radius and height of ceramic bulks, respectively). For LS, it was calculated by LS = (10–2*r*)/10.

### Extracts preparation

Based on the protocol of International Organization for Standardization (ISO 10993-5: 2009) [35], the extracts of Mg-contained CPS were prepared. Concisely, the powders of Mg-CPS were soaked in α-MEM (α-Minimum Essential Medium; HyClone, Logan, UT, USA) culture medium at the ratio of 200 mg ml^−1^ and then incubated for 24 h at 37°C. After the mixture was centrifugated for 10 min at the speed of 1600 rpm/min, the supernatants were collected and were filtered by 0.22 μm filter. Finally, the filtered supernatants were added with 10% fetal bovine serum (FBS; Gibco, Thermo Fisher Scientific, Waltham, MA, USA), 100 U ml^−1^ penicillin and 100 μg ml^−1^ streptomycin. The extracts were kept in refrigerator at 4°C. To illustrate the feature of Mg-CPS extracts, the apparent color, pH value (measured with digital pH meter) and ionic concentration (measured with ICP-OES, Agilent 725, Agilent Technologies, USA) of obtained extracts were analysed, respectively.

### Mc3t3-E1 cells and HUVECs culture

MC3T3-E1 mouse pre-osteoblast cell line (Subclone 14, ATCC, CRL-2594) and HUVECs (ATCC, PCS-100-010) were cultured in α-MEM medium containing 10% FBS, 100 U ml^−1^ penicillin and 100 μg ml^−1^ streptomycin. The medium was changed every other day. When confluence reached 80%, cell passing was conducted. Cells were cultured in 5% CO_2_ humidified incubator with temperature set at 37°C.

### Cell viability

To assess the proliferation of MC3T3-E1 cells and HUVECs stimulated by Mg-CPS extracts, the cell counting kit (CCK-8; Dojindo, Kumamoto, Japan) was used to detect the value of optical density (OD) at day 1, 4 and 7. Briefly, 3000 cells were seeded into each well of 96-well plates and were cultured with α-MEM medium for 24 h. On the next day, α-MEM was substituted by Mg-CPS extracts with 100 ml per well. The extracts were changed every other day. OD value was measured by the automatic microplate reader (TECAN, Männedorf, Switzerland), the absorbance/reference wavelengths was set as 450/630 nm. After that, Calcein/PI Cell Viability/Cytotoxicity Assay Kit (480/570) (Beyotime, Shanghai, China) was used and cells were analysed by fluorescence microscope (NIKON ECLIPSE TI-SR, Tokyo, Japan).

### Alkaline phosphate activity analysis

Concisely, the suspension of MC3T3-E1 cells were seeded into 6-well plates with 2 ml each at the concentration of 0.5 × 10^5^/ml. On the following day, α-MEM medium was replaced by the extracts supplemented with 10 nM β-glycerophosphate, 0.25 mM ascorbic acid and 20 nM dexamethasone. Every 3 days, the extracts were changed. On day 7 and 14, MC3T3-E1 cells were washed twice by phosphate buffer saline (PBS) and then were lysed by RIPA Lysis Buffer (Beyotime, Shanghai, China). After that, the supernatants were centrifugated for 5 min at 1 × 10^4^ rpm and were eventually collected. Alkaline phosphate (ALP) activity was calculated according to the instruction of Alkaline Phosphatase Assay Kit (Beyotime, Shanghai, China). Based on the protocol, total protein of MC3T3-E1 cells was calculated by Detergent Compatible Bradford Protein Assay Kit (Beyotime, Shanghai, China).

### Alizarin red staining

Briefly, MC3T3-E1 cell (0.5 × 10^5^/ml) suspension was seeded into 24-well plates with 1 ml per well for attachment. After 24 h, α-MEM medium was substituted by extracts supplemented with 10 nM β-glycerophosphate, 20 nM dexamethasone and 0.25 mM ascorbic acid. The medium was refreshed every 2 days. On day 14 July 2021, Alizarin red staining (Cyagen, Santa Clara, CA, USA) was used to observe mineralization status of MC3T3-E1 cells. Pictures were taken by light microscope and digital camera.

### Tube formation assay

Tube formation assay was applied to appraise angiogenic activity of HUVECs. Briefly, 200 μl of Cultrex^®^ Reduced Growth Factor Basement Membrane Extract (Cultrex^®^BME; Trevigen, Gaithersburg, MD, USA) was added into each well of 48-well plate and then the plate was placed in the 37°C incubator for 30 min. After the solidification of Cultrex^®^BME, 5 × 10^4^ HUVECs which have been cultured in every group of extract for 3 days were gently seeded into each well. Seeded HUVECs were incubated at 37°C for 12 h. Finally, photos were taken randomly by light microscope and analysed by using ImageJ containing the Angiogenesis Analyzer plugin (NIH, Bethesda, MD, USA).

### Quantitative real-time polymerase chain reaction assay

To evaluate osteogenic and angiogenic gene expression, quantitative real-time polymerase chain reaction (qRT-PCR) assay was applied. Concisely, MC3T3-E1 cells/HUVECs were seeded in a 6-well plate with 1 × 10^5^ each well and cultured with α-MEM medium for 24 h. On the following day when cells were attached, α-MEM medium was replaced with the extracts containing 10 nM β-glycerophosphate, 0.25 mM ascorbic acid and 20 nM dexamethasone. The extracts were refreshed every 2 days. For MC3T3-E1 cells, qRT-PCR analysis was conducted on day 7/14; for HUVECs, it was done on day 1/3. First, total RNA was extracted by utilizing the RNeasy mini kit (Qiagen, Duesseldorf, Germany); second, complementary DNA (cDNA) was reverse-transcribed by using all-in-one cDNA Synthesis Supermix (Bimake, Houston, TX, USA). Then the 7500 Real-time PCR system (Applied Biosystems; Thermo Fisher Scientific, Waltham, MA, USA) was operated to implement qRT-PCR analysis. CPS was set as control group and GAPDH was set as house-keeping gene. The reaction condition was followed by the pattern consisting of 95°C for 35 s and then 40 cycles of 95°C for 15 s and 60°C for 45 s. Gene expression was assessed via 2^–△△CT^ formula. The gene sequences were searched in GeneBank and listed in Supplementary Table S1.

### Enzyme linked immunosorbent assay

To calculate the amount of VEGF secreted by HUVECs, enzyme linked immunosorbent assay (ELISA) was applied on day 1/3. Concisely, 5 × 10^4^ HUVECs were placed into each well of a 6-well plate and cultured in extracts. After culturing for 1 or 3 days, each group’s supernatant was collected and quantitative concentration of VEGF was calculated by ELISA kit (R&D Systems, Minneapolis, MN, USA) based on the instructions within. The concentration was illustrated as ng/l.

### Western-blot analysis

Western-blot analysis was utilized to evaluate the expressions of intracellular cytokines in MC3T3-E1 cells and HUVECs. Having accomplished the course of culturing in the extracts, cells were rinsed twice by PBS and then were lysed by RIPA Lysis Buffer (Biotech, Shanghai, China). The concentration of protein was determined via using BCA Protein Assay Kit (Biotech, Shanghai, China), and then protein was electrophoresed through SDS-PAGE gels and transferred onto nitrocellulose membranes. The transfer membranes were then blocked with 5% BSA for 2 h in room temperature, washed by Tris Buffered saline Tween (TBST) for 5 min at three times and then incubated with primary antibodies overnight at 4°C. The membranes were washed by TBST for three times, incubated with horseradish peroxidase-conjugated secondary antibodies at room temperature for 2 h and then rinsed by TBST for 10 min at three times. The immunoreactive bands were visualized by the Odyssey infrared imaging system (LI-COR; Lincoln, Nebraska, USA). The intensity of bands was analysed by ImageJ software (NIH, Bethesda, MD, USA). The information of primary and secondary antibodies was listed in Supplementary Tables S2 and S3.

### Immunofluorescence

Immunofluorescence staining was conducted to evaluate the expression of angiogenic proteins (CD31/VEGFR1) on the membrane of HUVECs. Briefly, after placing one coverslip in each well of 6-well plate, 0.5 × 10^4^ HUVECs were seeded and cultured in α-MEM medium. After 6 h when HUVECs were attached on the coverslip, α-MEM medium was replaced by extracts. Having been cultured in extracts for 3 days, HUVECs were rinsed twice by PBS and were fixed by 4% paraformaldehyde for 20 min. Subsequently, CD31 (Abcam, Cambridge, UK) and VEGFR1 (Abcam) antibodies were used to assess CD31/VEGFR1 expression. Photos were taken by the inverted fluorescence microscope (NIKON ECLIPSE TI-SR, Tokyo, Japan).

### Statistical analysis

All the date above obtained from each group came from three samples at least, and each experiment was carried out three times. Data were exhibited as mean ± standard error of mean (SEM) and analysed by Two-way ANOVA through Tukey’s multiple comparisons test. Statistical analysis was performed by GraphPad Prism 7.0 (San Diego, CA, USA) software. If *P* < 0.05, statistical difference was considered significant.

## Results

### Composition and microstructure of Mg-CPS bioceramics

**Figure 1.**
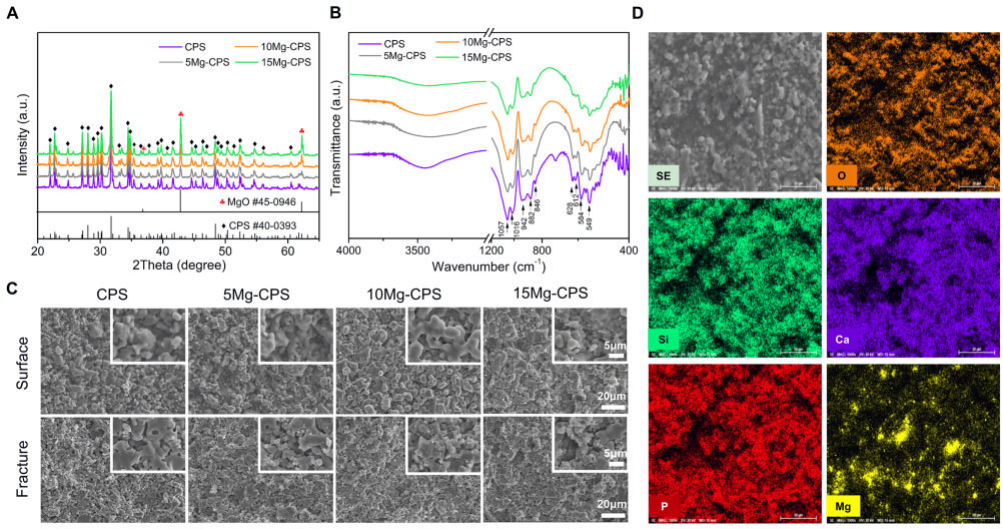
XRD patterns (**A**), FTIR spectra (**B**), and surface and fracture SEM images (**C**) of Mg-CPS bioceramics with different MgO contents and element mapping of 15 Mg-CPS (**D**)

XRD patterns of Mg-CPS bioceramics with different MgO contents sintered at 1300°C are shown in Fig. 1A. It is clear that the main diffraction peaks of all Mg-CPS specimens were indexed well to silicocarnotite (CPS, PDF# 40-0393), and the characteristic peaks of MgO (PDF# 45-0946) could also be detected and gradually increased with the addition of MgO nano-particles, meanwhile no other inferior Mg-containing phase appeared. FTIR spectra of Mg-CPS bioceramic powders were shown in Fig. 1B. The spectra exhibited characteristic peaks of CPS (1057 cm^−1^ ∼ 848 ${\text{cm}}_{,}^{-1}$ 628 cm^−1^ ∼ 549 cm^−1^) [36], while their intensity decreased along with MgO addition. The MgO stretching vibration band appeared at 430 ∼ 670 cm^−1^ range and the broad band at around 3500 cm^−1^ were attributed to stretching frequency of H-O-H.

Surface and fracture microstructural morphologies of Mg-CPS bioceramics are shown in Fig. 1C, displaying decreased porosity of ceramics with increased MgO content. While, the RD and LS of Mg-CPS bioceramics with increased MgO content have not changed dramatically (Supplementary Fig. S2), which means no remarkable accelerated sintering for CPS occurred like adding CuO [37], ZnO [31] and Fe_2_O_3_ [38] additives. The added MgO nano particles were likely just accumulated and compacted at the grain boundaries of CPS matrix. According to the element mapping results showed in Fig. 1D, it could be found that bright independent and dispersive particles were Mg-containing phases. On the contrary, the distribution of Ca, P and Si were complementary to Mg, which implied that no obvious reactions occurred between the main phase CPS and MgO addition.

### Mg-incorporation facilitated alkaline environment and Mg ion release in the extracts

**Figure 2.**
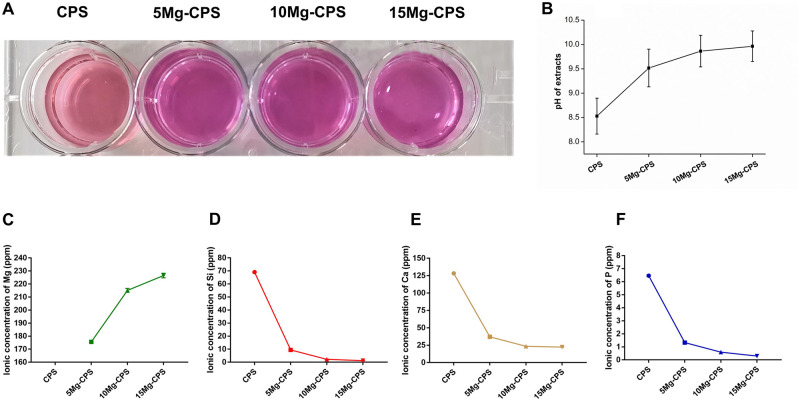
Characterization of Mg-CPS extracts. (**A** and **B**) Showed the color and pH of extracts. (**C**–**F**) Presented the ionic concentration of Mg, Si, Ca and P in extracts

As illustrated in Fig. 2A, the color of extracts became redder in appearance with increased MgO addition, particularly, the distinction of coloration between CPS and Mg-doped CPS was significant. Based on this, pH determination was introduced to measure the value and we found that the pH value enhanced with the improvement of mass fraction of MgO. Namely, the pH values of CPS, 5 Mg-CPS, 10 Mg-CPS and 15 Mg-CPS extracts were 8.53, 9.52, 9.86 and 9.96 (Fig. 2B). The element concentration of extracts was tested and shown in Fig. 2C– F. After 24-h soaking in α-MEM culture medium of Mg-CPS ceramic powders, Mg, Si, Ca and P elements released from Mg-CPS ceramic powders, which played important roles to the cell activities. While, their changing trends vs Mg content were different. Compared with CPS, Mg concentration elevated with the increase of MgO doped in CPS. However, the concentration of Si, Ca and P dramatically decreased with the addition of MgO, and further decreased slightly with the increase of MgO content. When MgO reached 15 wt.%, apart from the lowest concentration of Ca (22.4 ppm), Si and P were only 1.2 ppm and 0.3 ppm, respectively.

After extraction in α-MEM for 24 h, the Mg-CPS powders were filtered and their morphology and phase composition were tested by SEM and XRD respectively, which were displayed in Supplementary Fig. S3. Based on SEM morphology results, it was obvious to note that many tiny apatite crystals deposited on the surface of all three Mg-CPS powders, while, only a few apatite was observed on pure CPS case. Besides, from XRD patterns it could be found that after soaking, the diffraction intensity of both CPS and MgO peaks decreased evidently for all powders.

### Mg-incorporation overcame alkaline adversity and promoted the proliferation of MC3T3-E1 cells and HUVECs

**Figure 3.**
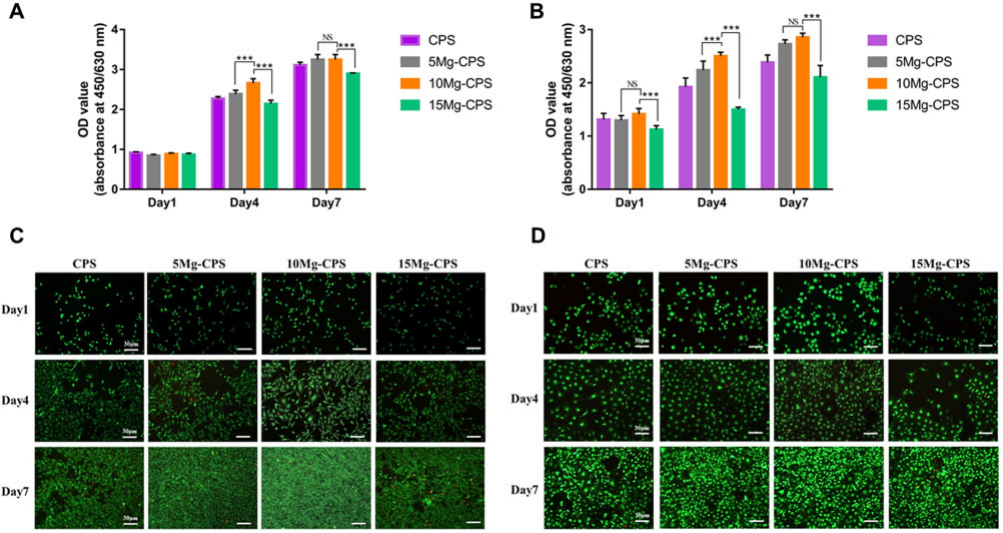
Proliferation of MC3T3-E1 cells (**A**) and HUVECs (**B**) cultured with extracts as well as live/dead cell staining of MC3T3-E1 cells (**C**) and HUVECs (**D**). (***P < 0.001, NS = not statistical)

As shown in Fig. 3, both MC3T3-E1 cells and HUVECs proliferated over culture time, but both groups exhibited dose-dependent effects among CPS, 5 Mg-CPS and 10 Mg-CPS. With the increasing dosage of MgO, OD values heightened. Especially from day 4, the OD value of 10 Mg-CPS was significantly higher than those of other three groups. On day 7, although there was no statistical difference in OD value between 5 Mg-CPS and 10 Mg-CPS, they were both significantly higher than that of pure CPS (*P* < 0.01). On the contrary, OD values of 15 Mg-CPS is lower than those of other groups on day 4 or day 7. For MC3T3-E1 cells (Fig. 3A), OD values were 2.27 and 2.14 in CPS and 15 Mg-CPS on day 4 (*P* < 0.01), 3.11 and 2.90 on day 7 (*P* < 0.001). For HUVECs (Fig. 3B), OD values were 1.92 and 1.50 in CPS and 15 Mg-CPS on day 4 (*P* < 0.01), 2.47 and 1.83 on day 7 (*P* < 0.001). Together with Live/Dead staining images, it illustrated that MC3T3-E1 cells and HUVECs steadily proliferated since the cell density grew with scarce death in CPS, 5 Mg-CPS and 10 Mg-CPS (Fig. 3C and D). In accordance to ISO-10993-5:2009(E) –8.5 [35], the extracts of CPS, 5 Mg-CPS and 10 Mg-CPS were non-toxic (Grade 0) and promoted cell proliferation. As for the extract of 15 Mg-CPS, despite its relatively slower growth and denser distribution of dead cells (Fig. 3C and D), OD value increased over culture time and thereby was considered slight toxicity (Grade 1).

### At the gene level, Mg ions upregulated osteogenic and angiogenic gene expressions in alkaline environment

**Figure 4.**
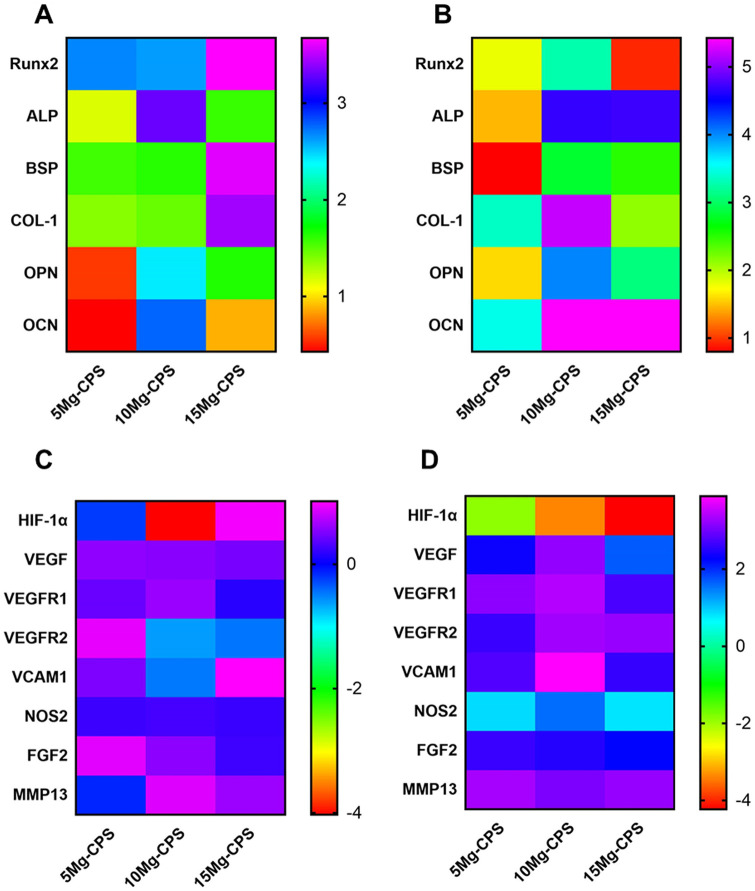
Fold increases of gene expression. (**A** and **B**) Illustrated osteogenic gene expression of MC3T3-E1 cells on day 7 (A) and day 14 (B). (**C** and **D**) Showed angiogenic gene expression of HUVECs on day 1 (C) and day 3 (D)

At the gene level, qRT-PCR was taken to measure relevant gene expressions under the combined impacts of elements and alkaline environment. It could be seen that except hypoxia inducible factor-1α, all genes had upregulated expression as culture time extended. Especially at day 14 for MC3T3-E1 cells (Fig. 4B) and at day 3 for HUVECs (Fig. 4D), gene expressions of 10 Mg-CPS were higher than those of 5 Mg-CPS (all *P* < 0.001). However, the increasing trend had somehow declined in 15 Mg-CPS, the group containing highest Mg^2+^ concentration (226.5 ppm) but strongest alkaline pH (pH = 9.65). Compare with 10 Mg-CPS (Mg^2+^ =215.1 ppm and pH = 9.54), the increasing fold of *Runx2*, *COL-1* and *OPN* was, respectively, 0.93, 2.12 and 3.09 in 15 Mg-CPS for MC3T3-E1 cells at day 14, significantly lower than 10 Mg-CPS at the same time (3.27 in *Runx2*, 5.22 in *COL-1* and 4.01 in *OPN*, all *P* < 0.001). As for HUVECs, even though there were not statistical differences in some gene expressions between 5 Mg-CPS and 10 Mg-CPS (*P* = 0.40 in *VEGFR1*, *P* = 0.74 in *FGF2* and *P* = 0.13 in *MMP13*) at day 3, the fold of 15 Mg-CPS had started decreasing at this time point. Namely, in comparison with the expressions of *VEGF*, *VEGFR1*, *VCAM1* and *NOS2* of 10 Mg-CPS (3.26-fold, 3.42-fold, 3.87-fold and 1.52-fold), statistical differences of fold were observed in 15 Mg-CPS (3.26 vs 1.64, *P* < 0.001 in *VEGF*; 3.42 vs 2.70, *P* = 0.02 in *VEGFR1*; *3.87* vs *2.78*, *P* < 0.001 in *VCAM1*; 1.62 vs 0.76, *P* < 0.01 in *NOS2*). The data of fold changes was listed in Supplementary Tables S4 and S5.

### At the protein level, enhanced osteogenesis of Mg-CPS through activating Smad2/3-Runx2 signaling pathway could be weakened in alkaline condition

**Figure 5.**
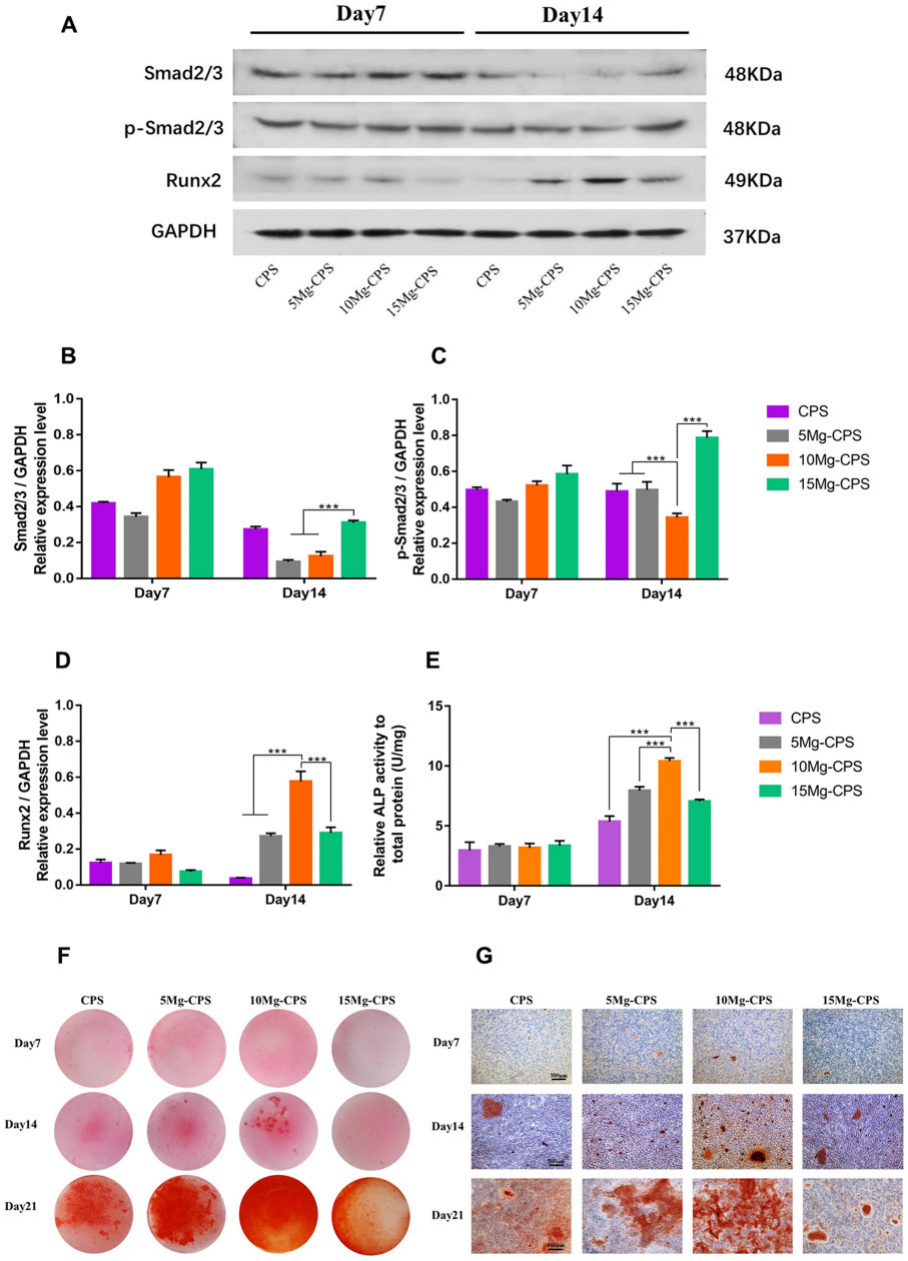
Expression of osteogenic proteins and mineralization. (**A**) Exhibited the levels of Runx2, Smad2/3, phosphorylated Smad2/3 (p-Smad2/3, activated form of Smad2/3) and GAPDH of MC3T3-E1 cells on day 7 and day 14. (**B**–**D**) Denoted the average ratios of Smad2/3/GAPDH (B), p-Smad2/3/GAPDH (C) and Runx2/GAPDH (D) according to the intensity analysis of gray bands. (***P < 0.001). (**E**) Showed relative ALP activity of MC3T3-E1 cells on day 7 and day 14. (**F** and **G**) Presented the alizarin red staining based on gross view and microscopic view magnified at 100× (scale bar =300 μm)

The effects of Mg^2+^ and alkaline pH on Mg-CPS osteogenesis were further investigated by western-blot and ALP analysis. By analysing the relative expression of Smad2/3-Runx2 signaling pathway and ALP activity at day 7 and day 14, it was found that as culture time lengthened, significant differences became evident, especially the comparison of 10 Mg-CPS and 15 Mg-CPS at day 14. According to the results of western-blot (Fig. 5A–C), Smad2/3 and p-Smad2/3 had both generally downregulated from day 7 to day 14 for CPS, 5 Mg-CPS and 10 Mg-CPS as differentiation time extended, but p-Smad2/3 of 15 Mg-CPS upregulated in the same period. We then explored the relative expression of Runx2, the transcript factors vital to modulating osteogenesis, and it had expressed highest in 10 Mg-CPS at day 7 and continued the trend as well at day 14 (Fig. 5D). It was obvious that Runx2 upregulation was accompanied with suppressed Smad2/3 expression over 14 days. Based on western-blot analysis, ALP activity analysis was adopted. As is presented in Fig. 5E, ALP activity increased in accordance with MgO addition to CPS, reaching the maximum when MgO was 10 wt.%, however, the trend reversed ranging from 10 wt.% to 15 wt.% (*P* < 0.001). Alizarin Red staining was further conducted to observe the osteogenic differentiation under the influence of Mg and alkalinity combined, and results were coherent with previous ones, illustrated as calcium nodules was distributed most densely in 10 Mg-CPS at day 14 and day 21. Nevertheless, osteogenic differentiation had drastically subsided in 15 Mg-CPS (Fig. 5F and G), where there existed highest Mg concentration (226.5 ppm) but also the highest alkaline pH (pH = 9.65).

### The advantage of Mg-incorporation to upregulate proangiogenic cytokines through activating PI3K-AKT pathway could be offset by alkaline environment

**Figure 6.**
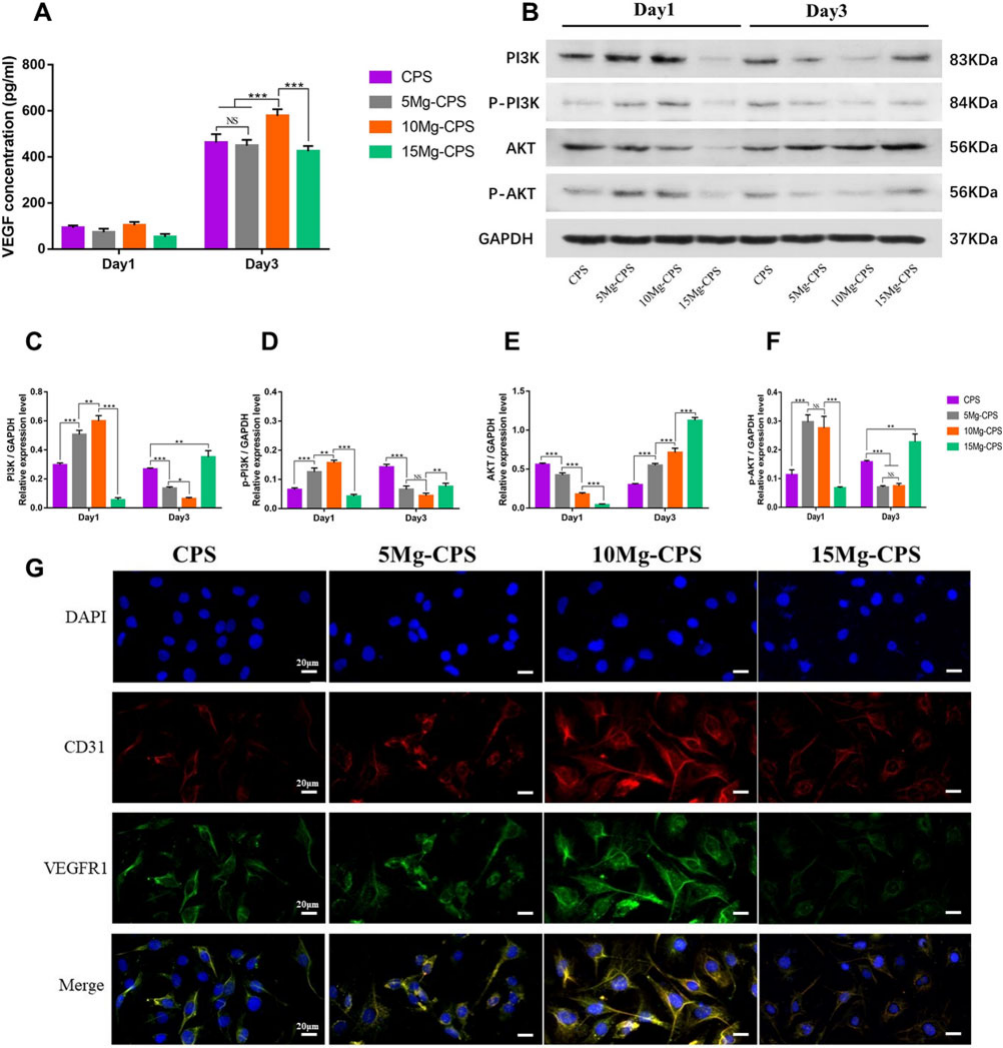
Expression and secretion of proangiogenic cytokines stimulated by extracts. (**A**) Exhibited the concentration of VEGF secreted by HUVECs in extracts on day 1 and day 3. (**B**) Showed the levels of PI3K, phosphorylated PI3K (p-PI3K, activated form of PI3K), AKT, phosphorylated AKT (p-AKT, activated form of PI3K) and GAPDH of HUVECs on day 1 and day 3. (**C**–**F**) Illustrated the average ratios of PI3K/GAPDH (C), p-PI3K/GAPDH (D), AKT/GAPDH (E) and p-AKT/GAPDH (F) based on the intensity analysis of gray bands. (*P < 0.05, ** *P* < 0.01, ***P < 0.001, NS = not statistical). (**G**) Presented the immunofluorescence of 4’,6-diamidino-2-phenylindole (DAPI) staining, anti-CD31 antibody, anti-VFGFR1 antibody and the merge of them (scale bar =20 μm)

To study the impacts of Mg-incorporation on angiogenic properties of CPS, we conducted ELISA, western-blot and immunofluorescence assay to analyse the changes of proangiogenic cytokines. First, ELISA was adopted to assess VEGF secretion into extracts, and although there was no difference at day 1, statistical difference was significant on 3-day consecutive culture in extracts. As shown in Fig. 6A, VEGF concentration of 10 Mg-CPS extract was 578.2 pg/ml on the third day, higher than any other group (*P* < 0.001), showing that Mg^2+^ could stimulate proangiogenic cytokine secretion despite the presence of alkaline pH. However, VEGF secretion decreased in 15 Mg-CPS, which contained highest Mg^2+^ concentration and the highest alkaline pH. Second, PI3K (phosphatidylinositol-3-kinase)-AKT (protein kinase B) pathway was explored to study intracellular changes caused by Mg-CPS extracts. It was discovered that except AKT, PI3K, p-PI3K and p-AKT had downregulated over time in CPS, 5 Mg-CPS and 10 Mg-CPS (Fig. 6B–F). Moreover, the three cytokines in 5 Mg-CPS and 10 Mg-CPS were expressed significantly higher than those in CPS at day 1(*P* < 0.001), but lower at day 3 (*P* < 0.001). It meant PI3K-AKT pathway was inhibited as culture time lengthened. Interestingly, the phenomena were in contrast to VEGF secreted in extracts (Fig. 6A), where the amount of VEGF increased as culture time extended and 5 Mg-CPS/10 Mg-CPS secreted more VEGF than CPS. As for 15 Mg-CPS where Mg and pH were both highest, PI3K-AKT pathway gradually activated and proangiogenic cytokines expressions were higher than 5 Mg-CPS or 10 Mg-CPS at day 3 (*P* < 0.01) (Fig. 6B–F). Third, we used immunofluorescence assay to evaluate the formation of proangiogenic proteins on the membrane of HUVECs (Fig. 6G). Moreover, the brightness extends of CD31 and VEGFR1 did not distinguish between CPS and 5 Mg-CPS. However, the brightness facilitated in 10 Mg-CPS shown as red and green rays were lighter than CPS or 5 Mg-CPS, and when MgO reached 15 wt.%, the brightness dwindled compared with 10 Mg-CPS.

### At the cellular movement level, the angiogenic efficacy of Si could be compensated and enhanced by Mg ions presence within proper alkaline pH

**Figure 7.**
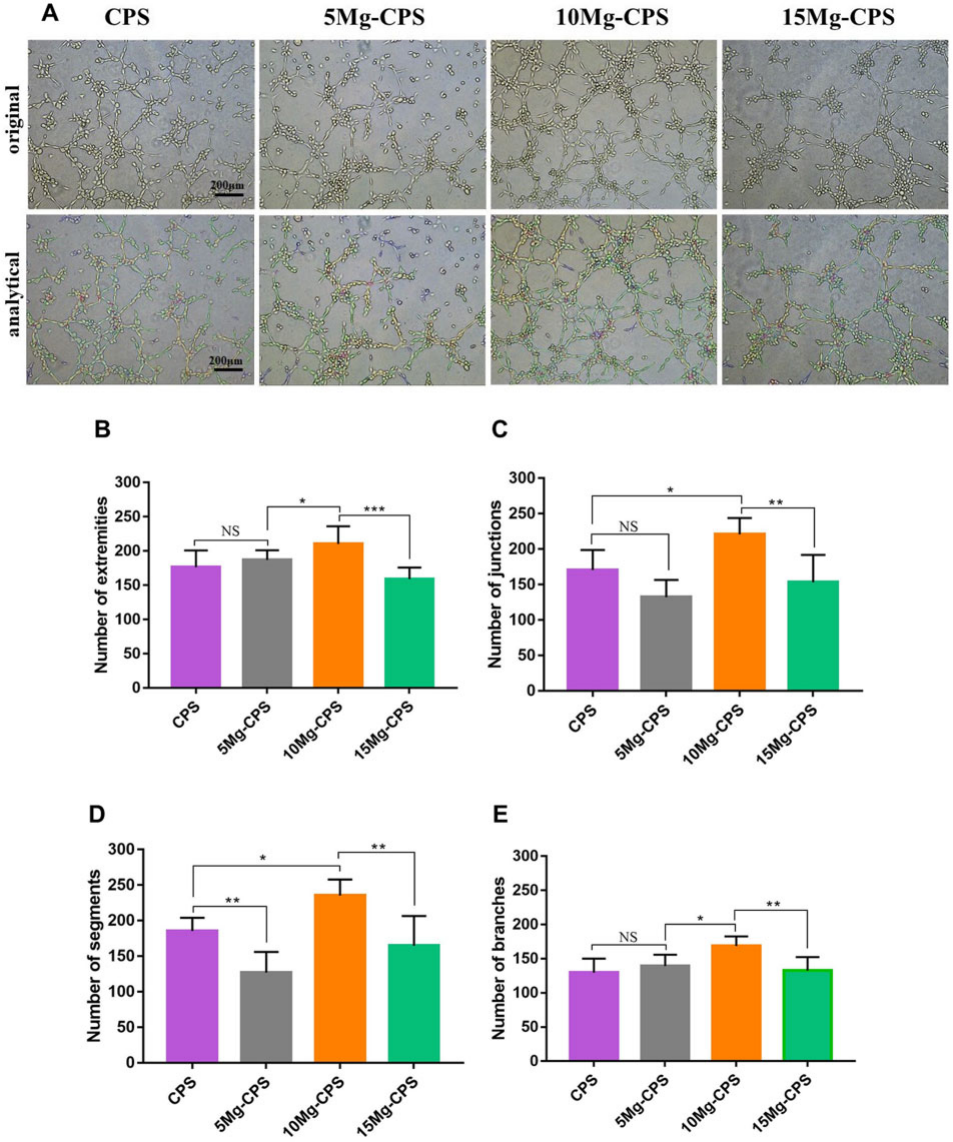
Tube formation assay of HUVECs cultured in extracts (**A**) and parameter analysis including numbers of extremities (**B**), junctions (**C**), segments (**D**) and branches (**E**). (*P < 0.05, ** *P* < 0.01, ***P < 0.001, NS = not statistical)

Tube formation assay was further conducted to assess angiogenic efficacy of HUVECs stimulated by the extracts (Fig. 7A), and representative parameters were adopted to analyse angiogenic ability. There were no statistical differences with regard to the numbers of extremities, junctions and branches between CPS and 5 Mg-CPS (*P* = 0.75, 0.09 and 0.74, respectively) (Fig. 7B, C and E). Considering the elements of CPS and 5 Mg-CPS extracts (Fig. 2C and D), it showed that the proangiogenic property of Si within CPS was analogous to the 5 wt.% MgO within CPS on the cellular level. And then, we found that as MgO content increased to 10 wt.% and pH reached 9.54, the numbers of four parameters of 10 Mg-CPS were significantly higher than those in any other group, denoting that proangiogenic character still improved. Nevertheless, if the mass fraction of MgO rose to 15 wt.%, the extract became the most alkaline amongst all groups. Synchronously, the angiogenic efficacy of 15 Mg-CPS compromised in spite of the highest Mg concentration.

## Discussion

Our study demonstrated the double-edged effects of Mg-CPS bioceramics on regulating osteogenic and angiogenic properties *in vitro*. Namely, although adding MgO into CPS was an efficient way to improve bioactivities, the inherent alkalinity caused by MgO would accompany and attenuate the benefit in the meantime. How to weigh the pros and cons is key to devising Mg-contained biomaterials, which is the purpose in our research.

Mg is the second abundant cation in cells and is essential to remain cellular homeostasis, especially regulating enzymes, ion exchange and signaling pathways [3, 39]. Besides, Mg homeostasis is important to stabilize mineral density and its disorder is often associated with osteoporosis [17, 40, 41]. Consequently, Mg has long been studied as a potential and promising orthopedic material in hope of promoting bone health [5, 9, 42, 43]. Particularly, degradable Mg-related metals is the main focus because released Mg^2+^ is able to stimulate osteogenic activity of bone marrow stromal cells and thus enhance bone regeneration [44–47]. However, as was pointed by Wang *et al*., degradation products of Mg and its alloys need to be evaluated carefully for standardization due to the cytotoxicity [48]. So developing new method is mandatory to objectively assess bioactivities and biocompatibilities of Mg-related biomaterials, especially physiochemical properties of extracts [49]. It is widely known that excessive alkaline environment caused by Mg and its alloys is cytotoxic via inhibiting cell viability and inducing hemolysis [21, 50–52], while material surface endowed with appropriate alkalinity can possess antibacterial and osteogenic properties [53, 54], so achieving the equilibrium of bioactivity and alkalinity is required to be considered when developing Mg-contained biomaterials.

In our study, MgO was adopted as a dopant to modify bioactive characters of CPS. From Fig. 1, it indicated that nano MgO addition did not react with the main phase CPS, and it dispersed independently and uniformly. When making extracts by immersing powders in culture medium for 24 h, MgO reacted with culture medium and formed Mg(OH)_2_. Since Mg(OH)_2_ is slightly soluble in water, so they mainly exist as Mg^2+^ and OH^-^ ions in extracts. Besides, the amount of formed Mg^2+^ and OH^-^ increased due to elevated MgO wt.% in CPS. As shown in Fig. 2A, the color of Mg-CPS extracts deepened compared with CPS because alkalinity intensified with MgO addition (Fig. 2B). Moreover, it also explained why Mg element had risen, because unlike the precipitation of Mg-CPS filtered during preparing extracts, Mg^2+^ could penetrate through the filter with 0.22 μm porosity (Fig. 2C). As for the decreased concentrations of Si, Ca and P with elevated MgO (Fig. 2D–F), it could be explained according to our previous finds that CPS had good apatite formation ability, besides, Ca^2+^, ${\text{PO}}_{4}^{3-}$ and ${\text{SiO}}_{4}^{4-}$ ions all participated this process [37], which would lead to the decrease of Ca, P and Si concentrations of extracts. Besides, the alkaline environment is more conducive to the formation of apatite [55], thus more tiny apatite crystals deposited on the surface of all three Mg-containing powders than pure CPS in higher pH environment (Supplementary Fig. S3), which may further demonstrate that Mg-CPS own superior bioactivity *in vitro* and potential osteogenic mineralization *in vivo*.

It is been confirmed that changing thickness of MgO films on implant materials could regulate alkalinity and prevent bacterial proliferation without having cytotoxic effects on osteoblast cells [56]. Based on this, we conducted CCK-8 and Live/Dead assay to further evaluate the effects of MgO concentration on cell viability (Fig. 3). It could be concluded that proper MgO incorporation (≤ 10 wt.%) was a feasible way to improve cell viability despite its concomitant alkalinity. Nevertheless, due to emerging alkaline cytotoxicity, the advantage of increasing Mg^2+^ was countered by the adverse impact of risen pH in 15 Mg-CPS extract, presented as relatively slower rate of cell proliferation (Fig. 3A and B) and evident distribution of dead cells on day 7 (Fig. 3C and D). Therefore, under the circumstance of existed double-edged effects, assessing osteogenic and angiogenic properties of Mg-CPS is also essential as the potential biomaterial.

In order to comprehensively understand changes in osteogenic activity caused by MgO incorporation, assays covering gene, protein and cellular levels were conducted. Mg is known to upregulate osteogenic genes in MC3T3-E1 cells and extracellular formation such as COL-1 in bone tissue [57]. It is therefore necessary to evaluate relevant expressions in our study. As PCR results showed (Fig. 4A and B), osteogenic gene expressions had all upregulated under the culture of Mg-CPS extracts regardless of Mg^2+^ and pH changes. Furthermore, Runx2 expression is worth studying because it is an important transcription factor responsible for other downstream gene regulations and osteogenic differentiation [58–60]. It was found that the trend of Runx2 changes in PCR was in consistent with its western-blot analysis (Fig. 5A and D), which meant that increasing Mg^2+^ outweighed grown alkalinity to promote Runx2 expression, thus causing the upregulation of other osteogenic genes on condition that MgO was within 10 wt.%. When MgO was 15 wt.% incorporated into CPS, the enhanced osteogenic activity was undercut by alkaline environment (pH > 10). In order to further prove it, the classical signaling pathway of Smad2/3-Runx2 was detected since its activation could enhance osteogenic differentiation [60, 61], and the results of our western-blot analysis showed that both Smad2/3 and p-Smad2/3 expressions were inhibited in CPS/5 Mg-CPS/10 Mg-CPS as culture period lengthened (Fig. 5B and C), which was opposite to Runx2 (Fig. 5D). That is because Runx2 protein is in the downstream of Smad2/3-Runx2 signaling pathway and directly stimulates other gene expressions by binding targeted DNA sequences [33]. Consequently, the feedback of Runx2 up-regulation inhibited Smad2/3 and p-Smad2/3 expression. It was the negative feedback that downregulated Smad2/3 phosphorylation. The explanation was also supported by the trend performance of 15 Mg-CPS, whose Runx2 expression was not as high as 10 Mg-CPS but Smad2/3 and p-Smad2/3 were both higher than it because of the weak negative feedback (*P* < 0.001). Apart from Smad2/3-Runx2 signaling pathway, we also conducted ALP activity assay because it is directly influenced by Runx2 and is vital of promoting osteoblastic differentiation and mineralization [62, 63], and the results (Fig. 5E) also matched the trend of Runx2 expression. In the end, Alizarin red staining was performed to observe the mineralization condition synchronously impacted by Mg^2+^ and alkaline environment (Fig. 5F and G), and the mineralization trend was consistent with Smad2/3-Runx2 signaling pathway and ALP activity. It should be reminded that A.M. Galow et al has reported alkaline pH could accelerate osteogenic differentiation and mineralization of MC3T3-E1 cells if it was within 8.4 [64]. Albeit more alkaline in Mg-CPS extracts, Mg^2+^ covered alkaline disadvantage to improve osteogenic differentiation if pH was within 10 in our study. So, the osteogenesis of Mg-CPS *in vitro* was final results of double-edged effects induced by Mg^2+^ and alkaline condition.

**Figure 8.**
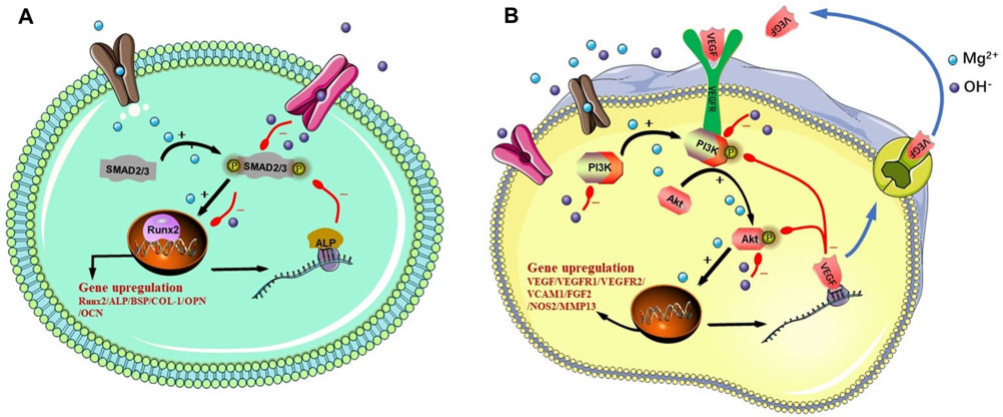
Signal pathways of Smad2/3-Runx2 in MC3T3-E1 cells (**A**) and PI3K-AKT in HUVECs (**B**)

Then the angiogenic changes of MgO incorporation was also studied based on three aspects including gene (PCR), protein (ELISA, western-blot and immunofluorescence) and cell (tube formation assay) levels. It was evident in our results that Mg^2+^ outperformed the adversity of strengthened pH if MgO was within 10 wt.%, resulting in the upregulation of proangiogenic gene expression (Fig. 4C and D) as well as elevated VEGF secretion in the extracts (Fig. 6A). Therefore, western-blot assay was taken to research the potential mechanisms leading to the changes of phenomena (Fig. 6B). PI3K-AKT pathway plays an important role in angiogenesis by facilitating endothelial cell activities [34, 65]. In particular, the activation of PI3K-AKT through phosphorylation is a key factor to contributing to endothelial cell movement [66]. Along with the fact that Mg^2+^ concentration within certain amount is able to upregulate angiogenic property and to stimulate VEGF secretion in HUVECs [15, 67], it is necessary to figure out the correlation between PI3K-AKT and Mg^2+^. Shown in the results, we found the interesting phenomenon that when Mg^2+^ concentration increased, VEGF concentration in extracts was in negative correlation with PI3K-AKT signaling pathway in HUVECs. On day 1 when secreted VEGF was low in extracts (Fig. 6A), PI3K-AKT had been activated by increased Mg^2+^ concentration, presented as enhanced relative expressions of PI3K, p-PI3K and p-AKT amongst CPS/5 Mg-CPS/10 Mg-CPS (Fig. 6C, D and F). For 15 Mg-CPS where there existed highest amount of Mg^2+^, PI3K-AKT was still inhibited on day 1 possibly due to stronger effect of alkalinity over Mg^2+^ concentration. On day 3, as 10 Mg-CPS secreted highest amount of VEGF in extracts (Fig. 6A), nevertheless, its relative expressions of PI3K, p-PI3K and p-AKT were lowest among CPS/5 Mg-CPS/10 Mg-CPS. It is probably because elevated synthesis of VEGF imposed negative feedback on PI3K-AKT pathway. It could be verified by 15 Mg-CPS as well, whose PI3K-AKT pathway gradually boosted during culture period because it was less negatively impacted by lower VEGF production compared with 10 Mg-CPS. We then conducted immunofluorescence to further confirm the double-edged effects induced by Mg^2+^ concentration and alkalinity combined. Both CD31 and VEGFR1, the classical markers of angiogenesis expressed on the membrane of HUVECs (Fig. 6G), exhibited similar trend with VEGF concentration in extracts. In the end, the cellular movement was evaluated because VEGF signaling regulates cell migration during vasculogenesis [68]. It could be seen that four parameters of tube formation assay were in accordance with VEGF amount secreted into extracts (Fig. 7). It was because VEGF mainly mediated the signals of intracellular and extracellular angiogenic cytokines, and they eventually affected endothelial cell migration [68, 69]. It was noteworthy in our study that the difference between CPS and 5 Mg-CPS was minimal, possibly because silicon ions released by CPS are also angiogenic by inducing VEGF expression [70]. Consequently, it resulted in similar VEGF secretion and tube formation assays in the two groups. In addition, angiogenic activities of HUVECs were upregulated by Mg^2+^ rather than suppressed by alkaline condition if MgO was incorporated within 10 wt.%. However, if MgO reached 15 wt.%, the environment consisting of higher-concentrated Mg ions, stronger alkalinity and lower Ca/P/Si ions caused resulted in declined angiogenic activities. In a word, promoted angiogenesis induced by Mg-CPS is the final outcome of Mg^2+^ and alkaline condition combined. The signal pathways in MC3T3-E1 cells and HUVECs were shown in Fig. 8.

## Conclusion

In our study, we demonstrate that MgO incorporation is a feasible way to improve osteogenic and angiogenic properties of CPS and that double-edged effects caused by MgO is the key reason to regulate bioactivities of Mg-CPS *in vitro*. Specifically, if MgO incorporation ranges from 0 wt.% to 10 wt.%, Mg^2+^ enhances osteogenesis of MC3T3-E1 cells via activating Smad2/3-Runx2 pathway, and also facilitates angiogenesis of HUVECs by upregulating PI3K-AKT signal. Under the circumstance, improved Mg^2+^ concentration in extracts compensates the adverse factor of increased alkalinity and therefore Mg-CPS bioceramics finally presents with advanced bioactivities. However, as MgO incorporation increases to 15 wt.%, the environment of higher-concentrated Mg ions, stronger alkalinity and lower Ca/P/Si ions caused will result in diminished bioactivities of Mg-CPS. Taken together, bioactivities of Mg-CPS are regulated by the double-edged effect induced by Mg ion concentration and alkaline condition *in vitro*. Provided that MgO is properly incorporated, the prospect of Mg-CPS bioceramics is hugely potential as a novel kind of orthopedic biomaterial for the osteogenic and angiogenic improvement. Moreover, impacted by the coupled consequences of Mg ion and alkalinity, the crosstalk between osteogenesis and angiogenesis is the promising field, which sheds light to the underlying mechanism in triggering bone tissue regeneration.

## Supplementary data

Supplementary data are available at *REGBIO* online.

## Funding

The work was supported by National Key R&D Program of China (No. 2018YFB1105600, No. 2018YFA0703000, No. 2017YFC1103800) and International Partnership Program of Chinese Academy of Sciences (Grant No. GJHZ1760).

## Author contributions

C.N. and K.D. conceived the idea and guided the writing; Q.W. designed the experiment, conducted *in vitro* experiments and wrote the article; S.X. designed the experiment, carried out the preparation and characterization of materials and wrote the article; F.W. and B.H. also contributed to related characterization of materials and X.W. and Y.S. offered instructions in writing the article.

*Conflict of interest statement*. None declared.

## Data availability

The data in this work are available in the manuscript or Supplementary Information, or available from the corresponding author upon reasonable request.

## Supplementary Material

rbab016_Supplementary_Data
