# Supplementary material for: Double-edged effects caused by magnesium ions and alkaline environment regulate bioactivities of magnesium-incorporated silicocarnotite in vitro
Source: Regen Biomater. 2021 Sep 2;8(6):rbab016. doi: 10.1093/rb/rbab016 (PMC8411036; doi:10.1093/rb/rbab016)
Supplement: rbab016_Supplementary_Data [file rbab016_Supplementary_Data.docx]

**Supplementary Data**

**Table S1. PCR Primers**

| **Full name** | **Abbreviation** | **GenBank No.** | **Sequences** |
| --- | --- | --- | --- |
| Mus musculus glyceraldehyde-3-phosphate dehydrogenase | mouse GAPDH | NM_001289726.1 | Forward: CCCTTAAGAGGGATGCTGCC  Reverse: ACTGTGCCGTTGAATTTGCC |
| Mus musculus runt related transcription factor 2 | Runx2 | NM_001271627.1 | Forward: TTCGCCTCACAAACAACCAC  Reverse: AACAAAACAAAACGGAGTGAGC |
| Mus musculus alkaline phosphatase | ALP | NM_007431.3 | Forward: GAACAGACCCTCCCCACGAG  Reverse: GTAGTCACAATGCCCACGGA |
| Mus musculus bone gamma carboxyglutamate protein | OCN | NM_007541.3 | Forward: GAACAGACAAGTCCCACACAGC  Reverse: TCAGCAGAGTGAGCAGAAAGAT |
| Mus musculus secreted phosphoprotein 1 | OPN | NC_000071.7 | Forward: AGGGCTATGGCTTGATCTCAC  Reverse: TGAACAGTAACGCCAAGGCT |
| Mus musculus integrin binding sialoprotein | BSP | NM_008318.3 | Forward: AGACAACGGAGAAGAAGCCG  Reverse: TCTCCCCCATACTCAACGGT |
| Mus musculus collagen, type I, alpha 1 | COL-1 | NC_000077.7 | Forward: ACAGTCGCTTCACCTACAGC  Reverse: AAGGCTAAACCAGATGCCCA |
| Homo sapiens glyceraldehyde-3-phosphate dehydrogenase | human GAPDH | NM_001256799.3 | Forward: GAAAGCCTGCCGGTGACTAA  Reverse: TTCCCGTTCTCAGCCTTGAC |
| Homo sapiens hypoxia inducible factor 1 subunit alpha | HIF1-alpha | NC_000014.9 | Forward: TTCACCTGAGGTAGGTGTCAT  Reverse: GTGCTCAGGTCTAGGTTAGGC |
| Homo sapiens vascular endothelial growth factor A | VEGF | NC_000006.12 | Forward: TTTTAAGGCCCCTGTGGTGG  Reverse: GTCTTGCCTCCCTGACTGAC |
| Homo sapiens vascular endothelial growth factor 1 | FLT1 | NM_002019.4 | Forward: GGGCTGAAACCATGTGCAAG  Reverse: GCCAAAGATGCACTCCTCCT |
| Homo sapiens vascular endothelial growth factor 2 | KDR | NM_002253.2 | Forward: GGCATGGGGTCTGTTCTGAA  Reverse: TTGGCCAGGAGACACGTAAC |
| Homo sapiens fibroblast growth factor 2 | FGF2 | NM_002006 | Forward: AAAAGGCAAGATGCAGGAGA  Reverse: TTTTGCAGCCTTACCCAATC |
| Homo sapiens nitric oxide synthase 2 | NOS2 | NM_000625.4 | Forward: ATGCTCAGCTCATCCGCTAT  Reverse: CGATGCACAGCTGAGTGAAT |
| Homo sapiens matrix metallopeptidase 13 | MMP13 | NM_002427 | Forward: TTTCAACGGACCCATACAGTTTG  Reverse: CATGACGCGAACAATACGGTTA |
| Homo sapiens vascular cell adhesion molecule 1 | VCAM1 | NM_001078 | Forward: CGTCTTGGTCAGCCCTTCCT  Reverse: ACATTCATATACTCCCGCATCCTTC |

**Table S2. Primary antibodies in Western-blot analysis**

| **Primary antibody** | **Abbreviation** | **Source** | **Dilution ratio** | **Manufacturing information** |
| --- | --- | --- | --- | --- |
| Runt-related transcription factor 2 | Runx2 | Rabbit | 1:1000 | Affinity Biosciences; Cincinnati, Ohio, USA |
| Mothers against decapentaplegic homolog 2/3 | Smad2/3 | Rabbit | 1:1000 | Affinity Biosciences; Cincinnati, Ohio, USA |
| Phosphorylated mothers against decapentaplegic homolog 2/3 | P-Smad2/3 | Rabbit | 1:1000 | Affinity Biosciences; Cincinnati, Ohio, USA |
| phosphatidylinositol 3-kinase | PI3K | Rabbit | 1:1000 | Affinity Biosciences; Cincinnati, Ohio, USA |
| Phosphorylated phosphatidylinositol 3-kinase | P-PI3K | Rabbit | 1:1000 | Affinity Biosciences; Cincinnati, Ohio, USA |
| AKT serine/threonine kinase | AKT | Rabbit | 1:1000 | Immunoway; Plano, Texas, USA |
| Phosphorylated AKT serine/threonine kinase | P-AKT | Rabbit | 1:1000 | Immunoway; Plano, Texas, USA |
| Glyceraldehyde-3-phosphate dehydrogenase | GAPDH | Mouse | 1:2000 | Biotech; Shanghai, China |

**Table S3. Secondary antibodies in Western-blot analysis**

| **Secondary antibody** | **Dilution ratio** | **Manufacturing information** |
| --- | --- | --- |
| Goat anti-Rabbit IgG (H+L) Cross-Adsorbed Secondary Antibody, HRP | 1:2000 | Thermo Fisher Scientific; Waltham, Massachusetts, USA |
| Goat anti-Mouse IgG (H+L) Cross-Adsorbed Secondary Antibody, HRP | 1:2000 | Thermo Fisher Scientific; Waltham, Massachusetts, USA |

**Table S4. Fold increases of osteogenic gene expression**

|  | **Day7** | | |  | **Day14** | | |
| --- | --- | --- | --- | --- | --- | --- | --- |
|  | **5Mg-CPS** | **10Mg-CPS** | **15Mg-CPS** |  | **5Mg-CPS** | **10Mg-CPS** | **15Mg-CPS** |
| Runx2 | 2.70±0.20 | 2.63±0.15 | 3.67±0.41 |  | 1.79±0.16 | 3.27±0.21 | 0.93±0.22 |
| ALP | 1.16±0.07 | 3.30±0.21 | 1.58±0.16 |  | 1.46±0.09 | 4.69±0.32 | 4.71±0.19 |
| BSP | 1.56±0.17 | 1.63±0.23 | 3.60±0.30 |  | 0.80±0.04 | 2.82±0.23 | 2.50±0.31 |
| COL-1 | 1.38±0.22 | 1.46±0.21 | 3.43±0.37 |  | 3.36±0.23 | 5.22±0.34 | 2.12±0.26 |
| OPN | 0.57±0.26 | 2.42±0.24 | 1.64±0.22 |  | 1.59±0.13 | 4.01±0.37 | 3.09±0.04 |
| OCN | 0.43±0.23 | 2.76±0.14 | 0.87±0.13 |  | 3.50±0.20 | 5.42±0.23 | 5.42±0.41 |

**Table S5. Fold increases of angiogenic gene expression**

|  | **Day1** | | |  | **Day3** | | |
| --- | --- | --- | --- | --- | --- | --- | --- |
|  | **5Mg-CPS** | **10Mg-CPS** | **15Mg-CPS** |  | **5Mg-CPS** | **10Mg-CPS** | **15Mg-CPS** |
| HIF-1α | -(0.30±0.16) | -(3.84±0.71) | 0.98±0.26 |  | -(1.83±0.22) | -(3.37±0.17) | -(4.03±0.40) |
| VEGF | 0.52±0.19 | 0.68±0.24 | 0.40±0.16 |  | 2.23±0.25 | 3.26±0.11 | 1.64±0.15 |
| VEGFR1 | 0.45±0.11 | 0.65±0.37 | 0.27±0.29 |  | 3.08±0.34 | 3.42±0.31 | 2.70±0.15 |
| VEGFR2 | 0.84±0.22 | -(0.43±0.33) | -(0.41±0.24) |  | 2.62±0.06 | 3.31±0.31 | 3.19±0.26 |
| VCAM1 | 0.48±0.07 | -(0.44±0.28) | 0.95±0.10 |  | 2.77±0.52 | 3.87±0.56 | 2.77±0.32 |
| NOS2 | 0.20±0.22 | 0.29±0.22 | 0.30±0.21 |  | 0.85±0.13 | 1.62±0.18 | 0.76±0.10 |
| FGF2 | 0.89±0.14- | 0.62±0.26 | 0.24±0.20 |  | 2.51±0.40 | 2.70±0.51 | 2.31±0.16 |
| MMP13 | -(0.16±0.04) | 1.06±0.36 | 0.62±0.08 |  | 3.28±0.22 | 2.76±0.62 | 3.34±0.37 |


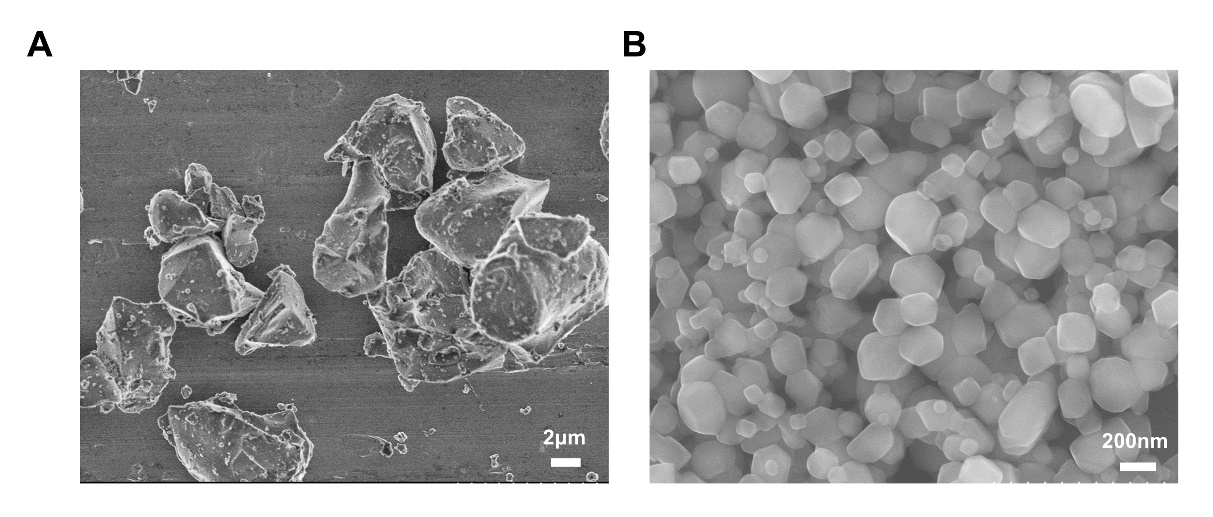


**Figure S1.** SEM morphologies of the staring powders: CPS (**A**) and MgO (**B**)


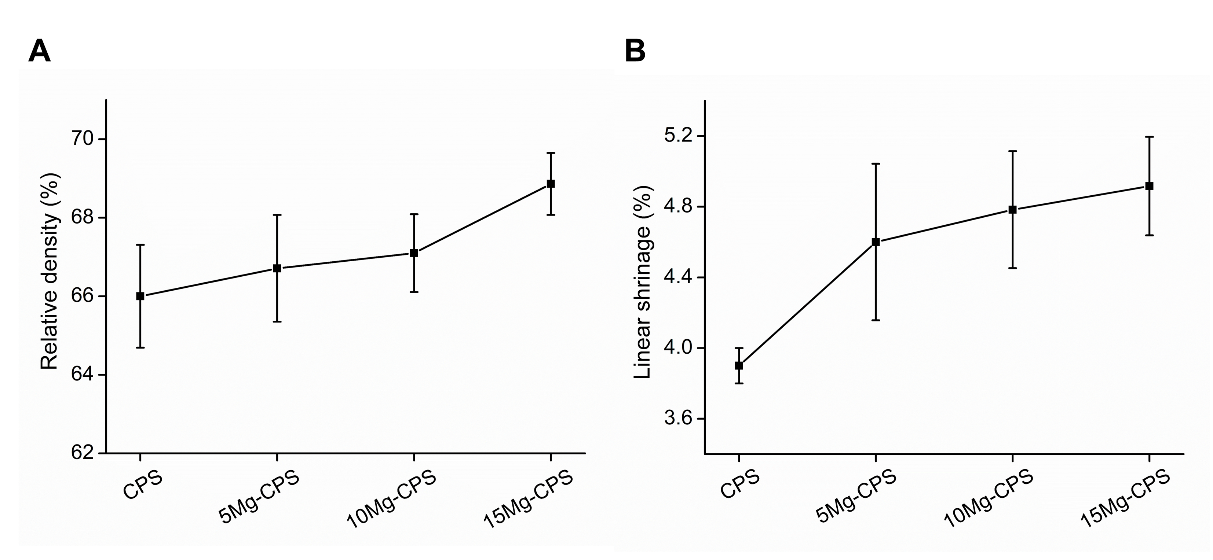


**Figure S2.** Relative density (**A**) and linear shrinkage (**B**) of Mg-CPS bioceramics with different MgO content


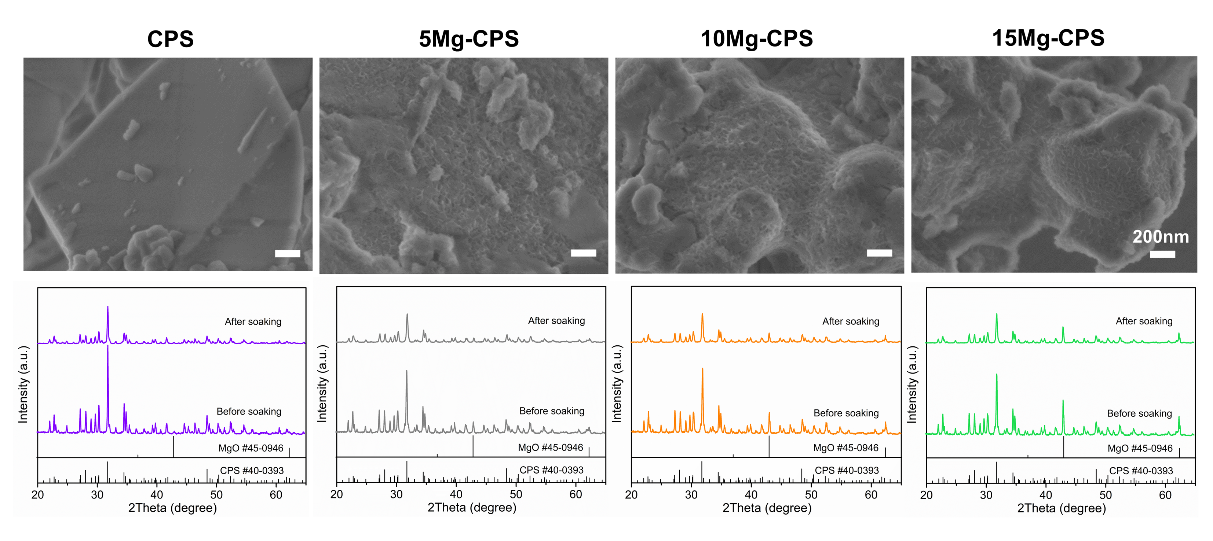


**Figure S3.** SEM morphologies and XRD patterns of Mg-CPS powders after soaking in α-MEM culture medium for extracts preparation
